# Evaluation of Propofol-Sparing Effect of Intravenous Lignocaine in Patients Undergoing Daycare Upper Gastrointestinal Endoscopic Procedures

**DOI:** 10.7759/cureus.32090

**Published:** 2022-12-01

**Authors:** Nidhi Arun, Mumtaz Hussain, Puja Kumari, Arvind Kumar

**Affiliations:** 1 Anaesthesiology, Indira Gandhi Institute of Medical Sciences, Patna, IND

**Keywords:** propofol-based sedation, anesthetic-sparing effect, daycare surgery, propofol based sedation, intravenous lignocaine infusion, gastrointestinal endoscopy

## Abstract

Background

Propofol is the most common sedative for endoscopies. Propofol alone may require larger doses for adequate level of sedation. Lignocaine is known for its anesthetic-sparing effect. We tested whether the addition of intravenous lignocaine to propofol-based sedation reduces its dose.

Methods

This prospective, randomized study was performed on 90 patients of 18 to 60 years of age, of either sex of the American Society of Anesthesiologists (ASA) Grade-I & II, and was divided into two groups. Group L + P received IV bolus of 1.5 mg/kg 2% lignocaine over 10 minutes followed by 1.5 mg/ kg/ h infusion and group NS + P- received the equivalent volumes of normal saline in bolus and infusion. Patients were induced with fentanyl (2 µg/kg) and propofol (1 mg/kg). To maintain an adequate sedation level, a supplemental bolus of 0.5 mg /kg propofol was administered. The outcomes recorded were the total and supplemental amount of propofol administered, as well as recovery time.

Results

The mean supplemental propofol for group L + P and group NS + P- 37.00 ± 29.93 and 58.67 ± 19.49 mg, respectively and mean total propofol consumption was 98.22 ± 34.00 mg and 131.11 ± 23.18 mg, respectively, (p < 0.001). Mean recovery time in group L + P was also shorter (5.22 ± 2.14 versus 9.96 ± 2.14). The incidence of adverse events like gag reflux, upper airway obstruction, pain on injection, and hypotension was significantly lower in group L + P (p < 0.05).

Conclusion

The addition of lignocaine to propofol-based sedation reduced the overall propofol requirement at the same time maintaining hemodynamic stability, spontaneous respiration, and early recovery.

## Introduction

Upper gastrointestinal (GI) endoscopies are commonly performed day care procedures. The most commonly used drugs are midazolam, propofol, or dexmedetomidine with or without short-acting opioids for sedation under monitored anesthesia care (MAC) to take care of patient’s anxiety and pain. Due to well-established hypnotic and sedative effect of propofol, it is widely used for MAC in endoscopic procedures [1]. Propofol is required in larger doses when used without any adjuvant for maintaining an adequate depth of sedation for endoscopy. Hypotension, upper airway obstruction, apnea, and abnormal reactions are the most common side effects of propofol when used in higher doses [2,3]. In our institute, fentanyl is routinely supplemented with propofol to reduce the dose of propofol by blunting endoscopy-induced sympathetic stimulation.

Lignocaine has proven anti-arrhythmic, analgesic as well as anti-nociceptive effects which contributes to anesthetic-sparing effect of lignocaine [4]. Other perioperative benefits of intravenous (IV) lignocaine include lower fatigue rate, shorter hospital stays, and the reduction of propofol injection pain [5-7]. Kranke et al. also concluded that perioperative lidocaine infusion at rates greater than or equal to 2 mg/kg/h was associated with decreased visual analog scale (VAS) pain scores, and opioid consumption in the first 24 h [8]. Lignocaine has been increasingly used as an adjuvant during general anesthesia due to its anesthetic-sparing effect. With the background of these known benefits of lignocaine, we have decided to use its infusion with propofol sedation for endoscopy to reduce the total propofol requirement, better patient recovery profile, and early discharge. To explore the pharmacokinetic interactions between these two drugs, Altermatt et al. have measured arterial propofol and lignocaine level and found that difference in the amount of propofol consumption is not due to changes in the concentration of arterial propofol. Thus, ruling out the pharmacokinetic interaction as a cause of propofol-sparing effect of lignocaine [9]. A randomized controlled study of 40 patients was conducted by Hans et al. to test the effect of IV lignocaine infusion on propofol requirement; they found that lignocaine decreased propofol requirements only during surgery. In the absence of surgical stimulation, lignocaine neither affects bi-spectral index (BIS) nor hemodynamic variables, establishing the anti-nociceptive property of lignocaine as a reason behind its hypnotic-sparing effect [10].

We conducted this investigation to test the hypothesis that a combination of infusion lignocaine and propofol to achieve adequate level of sedation for endoscopy reduces the total dose of propofol requirement while maintaining cardiovascular stability, spontaneous respiration, and early recovery.

## Materials and methods

A prospective, randomized, double-blinded study was performed in patients posted for upper GI endoscopic procedures under sedation over the period of 1 year after obtaining the approval of the Institute Ethics Committee (letter no. 1765/IEC/IGIMS/2020, dated 30/09/2020) and taking informed written consent (CTRI/2020/10/028697, Registered on: 28/10/2020). The calculated sample size was 90 keeping alpha error <0.05, beta error <0.2, and power of study <0.001. Ninety patients of 18 to 60 years of age, of either sex of the American Society of Anesthesiologists (ASA) Grade-I & II were randomly enrolled for this study. Patients with unstable hemodynamic status, mental disorders, refractory cancer pain, or allergic to study drugs were excluded.

After taking informed consent, patients were educated about the 11-point numerical rating score (NRS). Standard non-invasive monitoring like electrocardiogram (ECG), non-invasive blood pressure (NIBP), oxygen saturation (SpO_2_), and BIS were attached to the patients. Patients were randomly divided into two groups: Group L + P and group NS + P according to the random assignment generated through the computer. In group L + P, patients received IV bolus of 1.5 mg/kg 2% lignocaine over 10 minutes followed by 1.5 mg/ kg/ h continuous infusion of lignocaine, and in group NS + P-. Patients received the equivalent volumes of normal saline in bolus and infusion through infusion pump. Study infusions were prepared by anesthesia technicians involved neither in patient monitoring nor in collection of study variables. All patients in both groups were induced with fentanyl (2 µg/kg) and propofol (1 mg/kg) IV. All patients were asked for injection pain during the initial propofol administration. BIS values were achieved and maintained above 60. All patients were kept breathing spontaneously and oxygen was supplemented via nasal cannula at a flow rate of 4 L /min. The level of sedation was assessed every 5 minutes with the Modified Observer’s Assessment of Alertness/Sedation Scale (MOAA/S scale). The endoscope was inserted only after achieving MOAA/S score ≤1. Presence or absence of a gag event was noted at the time of the first attempt to the introduction of endoscope down the patient’s mouth. A gag event was defined as a gag reflex with involuntary movements. Bolus of propofol (0.5 mg/kg) was supplemented whenever MOAA/S score exceeded 1 or BIS value came above 60 to maintain an adequate level of sedation after induction. The possibility of bias from over-sedation was ruled out, as we employed both objectives as well as subjective sedation scores during the entire procedure in the form of BIS and MOAA/S scales. 

All medications were stopped once the endoscope was taken out at the end of the procedure. Those patients who needed airway management or converted to general anesthesia (GA), or when the duration of the endoscopic procedure exceeded 60 minutes, were excluded from the study. Post-procedure patients were shifted to the post-anesthesia recovery unit (PACU). Post-procedure pain scores were recorded by 11-point NRS, just after admission in PACU and then after 30 minutes. Endoscopist satisfaction score was asked and recorded on a 10-point VAS. The primary outcomes recorded were 1) total amount of propofol administered, 2) total amount of supplemental propofol after induction, 3) recovery time, 4) post-procedure pain score, and 5) endoscopist satisfaction score. Apart from this, the incidence of gag event and involuntary movements during the introduction of the endoscope and basic hemodynamic parameters like heart rate (HR), NIBP, SpO_2_ were recorded at 5 minutes interval and episodes of apnea or incidence of any other adverse events were recorded at different time points during endoscopy.

Statistical analysis

The data were recorded and analysis was done using EPI info (version 7.2). The qualitative data were shown in terms of percentages. The quantitative data were expressed either in percentages or in mean and standard deviations. Chi-square or Fisher exact test was used to analyze the differences between the two proportions. Student t-test was used to find out the difference between the two means. All analysis was two-tailed and p < 0.05 was considered significant.

## Results

**Figure 1:**
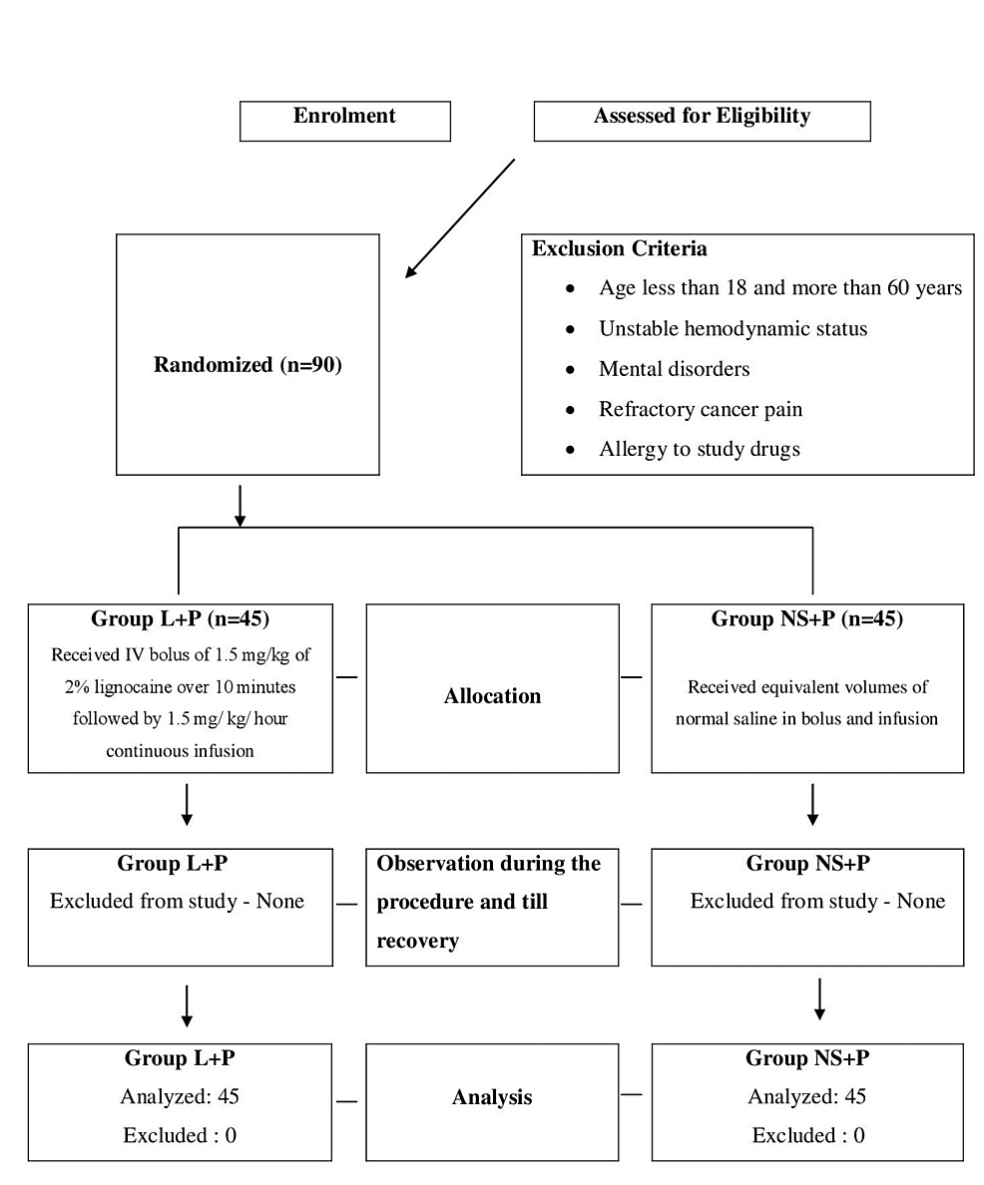
Simplified methodology of conduction of study shown as CONSORT diagram

Out of 90 patients fulfilling the inclusion criteria, we have randomly allocated 45 cases in each group (group L + P and group NS + P) (Figure 1).

Patients of both groups were demographically comparable (Table 1).

**Table 1:** Demographic parameter *ASA: American Society of Anesthesiologists

S. No.	Parameter	Group L + P (Mean ± SD)	Group NS + P (Mean ± SD)	P value
1.	Age (years)	43.76 ± 14.20	44.84 ± 14.01	0.7173
2.	Gender (female: male)	25:20	30:15	0.2796
3.	Weight (kg)	60.60 ± 13.65	62.16 ± 15.24	0.6136
4.	ASA* (grade I: grade II)	30:15	28:17	0.6596

**Figure 2:**
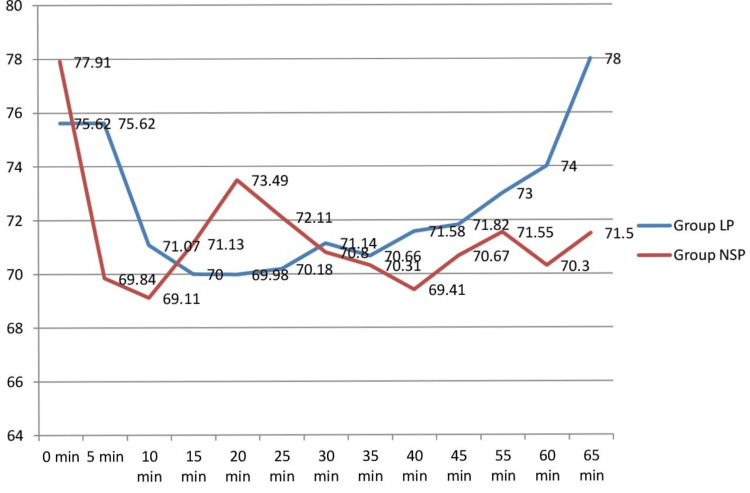
MAP* at different time intervals in both groups *MAP: mean arterial pressure

The mean age of the cases among group L + P was 43.76 ± 14.20 years and group NS + P was 44.84 ± 14.01 years and the difference was not statistically significant (p = 0.7173). There was no significant difference between the two groups based on gender (p = 0.2796), weight (p = 0.6136), and ASA physical status (p = 0.6596) of the patients. The hemodynamic parameters like NIBP, HR, and SpO_2_ remained within normal limits throughout the procedure in patients of both groups (Figure 2).

Mean duration of the endoscopic procedure was also comparable, 29.22±8.59 versus 28.11±6.51 minutes in group L + P versus NS + P, respectively (p = 0.4911). Patients of both groups were induced with similar doses of IV fentanyl and propofol. Propofol bolus (0.5 mg/kg) was supplemented IV when required during the procedure to keep the BIS value between 60 and 80 and MOAA/S score ≤1. When we calculated and compared the amount of supplemental propofol in both groups, we found that the mean supplemental propofol in group L + P was significantly less as compared to group NS + P (37.00 ± 29.93 and 58.67 ± 19.49 mg, respectively). As a result, total propofol consumption in group L + P during the whole procedure was also significantly less (p < 0.001). The calculated mean total propofol consumption in group L + P was 98.22 ± 34.00 mg and in group NS + P was 131.11 ± 23.18 mg. Recovery time was defined as time period between the end of the endoscopic procedure and the awake patient with MOAA/S score ≥ 2. Mean recovery time in minutes in group L + P was shorter (5.22 ± 2.14) than in group NS + P (9.96 ± 2.14). This difference was also found to be statistically significant (p < 0.001) (Table 2).

**Table 2:** Observations

S. No.	Parameter	Group L + P (Mean ± SD)	Group NS + P (Mean ± SD)	P value
1.	Duration of endoscopic procedure (min)	29.22 ± 8.59	28.11 ± 6.51	0.4911
2.	Supplemental propofol (mg)	37.00 ± 29.93	58.67 ± 19.49	<0.001
3.	Total propofol consumption (mg)	98.22 ± 34.00	131.11 ± 23.18	<0.001
4.	Recovery time (min)	5.22 ± 2.14	9.96 ± 2.50	<0.001

Post-procedure pain score was significantly lower in group L + P than in group NS + P (Table 3).

**Table 3:** Post-procedure pain score *NRS: numerical rating scale **PACU: Post-anesthesia care unit

S. No.	Parameter	Group L + P (Mean ± SD)	Group NS + P (Mean ± SD)	P value
1.	NRS* at the time of admission in PACU**	3.55±0.65	5.02±0.99	<0.001
2.	NRS after 30 minutes of admission in PACU	3.44±0.54	5.31±0.82	<0.001

Though no significant difference in pain score was found between NRS documented just after arriving in PACU and at 30 minutes after, in either group. Endoscopist satisfaction score assessed by 0-10 VAS score was comparable in both groups. Incidence of gag event on the first attempt of endoscopy was significantly higher (p = 0.006) in group NS + P (33.33%) than in group L + P (11.11%). Episodes of upper airway obstructions were identified by either paradoxical respiratory patterns or episodes of oxygen desaturation (SpO_2_ < 95%). The incidence of upper airway obstruction was documented to be significantly higher (p < 0.05) in group NS + P (31.11%) as compared to group L + P (8.89%). But none of them needed definitive airway management. These undesirable episodes were easily managed by jaw thrust maneuvers or use of nasal airway. The incidence of hypotension was lesser (13.33%) among group L + P as compared to patients belonging to group NS + P (22.22%), though this difference was not significant. Similarly, the incidence of bradycardia was also less in group L + P (13.33%) than in group NS + P (4.44%), but this difference was not statistically significant. Only a fewer patients (4.44%) in group L + P complained of pain on propofol injection, in comparison to 33.33% among group NS + P and this difference was statistically significant (p < 0.05) (Table 4).

**Table 4:** Incidence of adverse events

S. No.	Adverse event	Group L + P		Group NS + P		P value
		Number	%	Number	%	
1.	Gag event during first attempt of endoscopy	5	11.11	15	33.33	0.006
2.	Upper airway obstruction	4	8.89	14	31.11	0.0321
3.	Hypotension	6	13.33	10	22.22	0.1213
4.	Bradycardia	6	13.33	2	4.44	0.3211
5.	Pain on injection	2	4.44	15	33.33	0.0021

## Discussion

Our results demonstrated that peri-procedural infusion of lignocaine to the propofol during upper GI endoscopic procedure reduced supplemental as well as total propofol dosage requirements, post-procedure pain, and shortened recovery time without significant hemodynamic and respiratory adverse events. Propofol-associated adverse events are dose-dependent, when used in higher doses, the incidence of cardio-respiratory depression is more frequent. The propofol-sparing effect of IV lignocaine may reduce the risk of these complications, especially in the older population [11].

Because of the anti-nociceptive and analgesic properties of lignocaine, only 4.44% of patients receiving lignocaine infusion had pain on propofol injection. The incidence of hypotension, bradycardia, and upper airway obstruction, usually associated with the use of higher doses of propofol, was found to be lesser among group L + P, but this difference was not significant. There was no significant difference in the duration of endoscopic procedures between the two groups.

Several studies had also been done to see the effect of topical lignocaine in gastro-endoscopic procedures under conscious sedation with varied results. Few of them reported the effectiveness of topical lidocaine spray, but few failed to reproduce the result under similar settings. One meta-analysis revealed that topical lidocaine pharyngeal spray with sedation provided an optimal condition and better tolerance by patients to gastro-endoscopy. However, the included randomized controlled trials (RCTs) had obvious diversity, and propofol was not uniformly used for sedation [12]. We observed a lesser incidence of gag event in the group receiving IV lignocaine. Results of a meta-analysis of 12 RCTs evaluating the impact of topical as well IV lignocaine along with propofol sedation for gastro-endoscopic procedures, by Hung et al., demonstrated that topical lignocaine could decrease the risk of gag events, without reducing the propofol dosage requirement and a significant reduction in propofol dosage when IV lignocaine was supplemented. However, this reduction in propofol requirement was not associated with a decrease in the risk of hypoxemia or hypotension as compared with that of the placebo [13]. Therefore, we emphasize the importance of careful monitoring and oxygen supplementation in every patient posted for GI endoscopic procedures under sedation, even when smaller dosage of propofol is used.

A randomized controlled study of 40 patients was conducted by Hans et al. to test the effect of IV lignocaine infusion on propofol requirement; they found that lignocaine decreased propofol requirements only during surgery [13]. In the absence of surgical stimulation, lignocaine neither affects BIS nor hemodynamic variables, establishing the anti-nociceptive property of lignocaine as a reason behind its hypnotic-sparing effect [11]. We also found comfortable patients with significantly lesser post-procedure pain scores in patients receiving IV lignocaine with propofol. Another study also supported that propofol dosage was decreased by as much as 50% when IV lignocaine was used with propofol sedation for colonoscopy. A significant reduction in post-colonoscopy pain and fatigue was also seen [14]. Our study results also confirmed a reduction in the amount of supplemental propofol. Reduction in the requirement of supplemental propofol resulted in a significant reduction in amount of total propofol consumed in group receiving IV lignocaine infusion. Consequently, recovery time was also shorter in this group. Though there are studies where some patients receiving IV lignocaine had delayed recovery and extubation this can be explained by longer infusions of lignocaine and propofol in these studies [15-17].

As per the international consensus statement on efficacy and safety of the use of IV lignocaine, it should be regarded as a “high-risk” medicine and should be used with caution. Ideal body weight should be used for dose calculation. A loading dose of no more than 1.5 mg/kg over 10 min and an infusion of no more than 1.5 mg/kg/h, for no longer than 24 h is recommended and should be delivered from a suitable infusion device through a separate, dedicated cannula with separate lidocaine monitoring chart [18]. We followed all these guidelines while conducting this study. Administration of lignocaine as IV bolus followed by infusion was preferred to achieve a steady-state concentration while minimizing systemic toxicity. A bolus dose prior to a continuous infusion was given to accelerate the achievement of desired therapeutic concentration [19].

A recently reported large series showed a lower incidence of adverse events (6.8%) in whom IV lignocaine had been used [20]. In patients with hepatic/renal impairment, academia, hypoxemia, hypoalbuminemia, or in prolonged infusion, lignocaine metabolism and its clearance might get delayed and serum lignocaine level might reach a toxic level. The most commonly manifested minor adverse effects are drowsiness, light-headedness, perioral numbness, tinnitus, and bradycardia. Thus, patient selection, informed consent, and proper monitoring in the high-dependency unit should be done in patients receiving lignocaine infusion.

Our findings were similar to that of previous studies conducted to establish the beneficial outcomes of using IV lignocaine with propofol. These studies have suggested that IV lignocaine may improve post-procedure recovery such as early recovery of GI function, lower post-endoscopy pain, and fatigue scores, decreased nausea and vomiting, early recovery, and better endoscopist satisfaction score [21,22].

Our patients were of age between 18 and 60 years, belonging to ASA-I and II. The demographic profile of the study subjects was a major limitation of our study. Respiratory complications like hypoventilation and apnea are major concerns in older patients with multiple comorbidities [10]. Obese patients and history of obstructive sleep apnea (OSA) are significant risk factors for the development of hypoxia in a patient undergoing sedation for endoscopic procedures [23,24]. We didn’t observe any major respiratory adverse events in our study. Further studies are needed to evaluate the beneficial effects of supplementation of IV lignocaine with propofol in high-risk study samples.

## Conclusions

Propofol alone may require larger doses for an adequate level of sedation. The addition of lignocaine to propofol-based sedation reduced the overall propofol requirement at the same time maintaining hemodynamic stability, spontaneous respiration, and early recovery. Thus, we suggest the usage of IV infusion of lignocaine in recommended dose for a shorter duration as a propofol-sparing agent with careful monitoring and oxygen supplementation in patients undergoing sedation for upper GI endoscopic procedures. The recommended protocol may enhance endoscopy unit efficiency by shortening the recovery time.

## References

[1] Horiuchi A, Nakayama Y, Kajiyama M, Kato N, Kamijima T, Ichise Y, Tanaka N (2012). Safety and effectiveness of propofol sedation during and after outpatient colonoscopy. World J Gastroenterol.

[2] Egan TD (2019). Are opioids indispensable for general anaesthesia?. Br J Anaesth.

[3] Dossa F, Medeiros B, Keng C, Acuna SA, Baxter NN (2020). Propofol versus midazolam with or without short-acting opioids for sedation in colonoscopy: a systematic review and meta-analysis of safety, satisfaction, and efficiency outcomes. Gastrointest Endosc.

[4] Lee SH, Park YK, Lee DJ, Kim KM (2014). Colonoscopy procedural skills and training for new beginners. World J Gastroenterol.

[5] Herroeder S, Pecher S, Schönherr ME (2007). Systemic lidocaine shortens length of hospital stay after colorectal surgery: a double-blinded, randomized, placebo-controlled trial. Ann Surg.

[6] Yardeni IZ, Beilin B, Mayburd E, Levinson Y, Bessler H (2009). The effect of perioperative intravenous lidocaine on postoperative pain and immune function. Anesth Analg.

[7] Picard P, Tramèr MR (2000). Prevention of pain on injection with propofol: a quantitative systematic review. Anesth Analg.

[8] Kranke P, Jokinen J, Pace NL (2015). Continuous intravenous perioperative lidocaine infusion for postoperative pain and recovery. Cochrane Database Syst Rev.

[9] Altermatt FR, Bugedo DA, Delfino AE, Solari S, Guerra I, Muñoz HR, Cortínez LI (2012). Evaluation of the effect of intravenous lidocaine on propofol requirements during total intravenous anaesthesia as measured by bispectral index. Br J Anaesth.

[10] Hans GA, Lauwick SM, Kaba A (2010). Intravenous lidocaine infusion reduces bispectral index-guided requirements of propofol only during surgical stimulation. Br J Anaesth.

[11] Amornyotin S, Srikureja W, Chalayonnavin W, Kongphlay S (2011). Dose requirement and complications of diluted and undiluted propofol for deep sedation in endoscopic retrograde cholangiopancreatography. Hepatobiliary Pancreat Dis Int.

[12] Evans LT, Saberi S, Kim HM, Elta GH, Schoenfeld P (2006). Pharyngeal anesthesia during sedated EGDs: is "the spray" beneficial? A meta-analysis and systematic review. Gastrointest Endosc.

[13] Hung KC, Yew M, Lin YT (2022). Impact of intravenous and topical lidocaine on clinical outcomes in patients receiving propofol for gastrointestinal endoscopic procedures: a meta-analysis of randomised controlled trials. Br J Anaesth.

[14] Forster C, Vanhaudenhuyse A, Gast P, Louis E, Hick G, Brichant JF, Joris J (2018). Intravenous infusion of lidocaine significantly reduces propofol dose for colonoscopy: a randomised placebo-controlled study. Br J Anaesth.

[15] Nishino T, Hiraga K, Sugimori K (1990). Effects of i.v. lignocaine on airway reflexes elicited by irritation of the tracheal mucosa in humans anaesthetized with enflurane. Br J Anaesth.

[16] Steinhaus JE, Gaskin L (1963). A study of intravenous lidocaine as a suppressant of cough reflex. Anesthesiology.

[17] Bailey JM (1995). Technique for quantifying the duration of intravenous anesthetic effect. Anesthesiology.

[18] Foo I, Macfarlane AJ, Srivastava D (2021). The use of intravenous lidocaine for postoperative pain and recovery: international consensus statement on efficacy and safety. Anaesthesia.

[19] Bennett PN, Aarons LJ, Bending MR, Steiner JA, Rowland M (1982). Pharmacokinetics of lidocaine and its deethylated metabolite: dose and time dependency studies in man. J Pharmacokinet Biopharm.

[20] De Oliveira K, Eipe N (2020). Intravenous lidocaine for acute pain: a single-institution retrospective study. Drugs Real World Outcomes.

[21] Weibel S, Jokinen J, Pace NL (2016). Efficacy and safety of intravenous lidocaine for postoperative analgesia and recovery after surgery: a systematic review with trial sequential analysis. Br J Anaesth.

[22] Ko HH, Zhang H, Telford JJ, Enns R (2009). Factors influencing patient satisfaction when undergoing endoscopic procedures. Gastrointest Endosc.

[23] Wani S, Azar R, Hovis CE (2011). Obesity as a risk factor for sedation-related complications during propofol-mediated sedation for advanced endoscopic procedures. Gastrointest Endosc.

[24] Patel VA, Romain PS, Sanchez J, Fisher DA, Schulteis RD (2017). Obstructive sleep apnea increases the risk of cardiopulmonary adverse events associated with ambulatory colonoscopy independent of body mass index. Dig Dis Sci.

